# A flea and tick collar containing 10% imidacloprid and 4.5% flumethrin prevents flea transmission of *Bartonella henselae* in cats

**DOI:** 10.1186/1756-3305-6-26

**Published:** 2013-01-25

**Authors:** Michael R Lappin, Wendell L Davis, Jennifer R Hawley, Melissa Brewer, Arianne Morris, Dorothee Stanneck

**Affiliations:** 1 Department of Clinical Sciences, Center for Companion Animal Studies, Colorado State University, Fort Collins, USA; 2 Bayer HealthCare LLC, Shawnee, KS, USA; 3 Bayer Animal Health GmbH, Leverkusen, Germany

**Keywords:** Bartonella, Ctenocephalides, Imidacloprid

## Abstract

**Background:**

*Bartonella henselae* is transmitted amongst cats by *Ctenocephalides felis* and is associated with multiple clinical syndromes in cats and people. In a previous study, monthly spot-on administration of 10% imidacloprid/1% moxidectin was shown to block transmission of *B. henselae* amongst cats experimentally exposed to infected *C. felis*. The purpose of this study was to determine whether application of a flea and tick collar containing 10% imidacloprid and 4.5% flumethrin would lessen *C. felis* transmission of *B. henselae* amongst cats for 8 months.

**Methods:**

Specific pathogen free cats (n = 19) were housed in three adjoining enclosures that were separated by mesh to allow *C. felis* to pass among groups but prevent cats in different enclosures from contacting one another. One group of 4 cats was inoculated intravenously with *B. henselae* and after infection was confirmed in all cats based on positive PCR assay results, the cats were housed in the middle enclosure. The *B. henselae* infected cat group was flanked by a group of 8 cats that had the collar placed and maintained for the duration of the study and a group of 7 cats that were not treated. *Ctenocephalides felis* (50 males and 50 females) raised in an insectary were placed on each of the 4 cats in the *B. henselae* infected group monthly for 7 applications and then every 2 weeks for 4 applications starting the day the collar was applied. Blood was collected from all cats weekly for *Bartonella* spp. PCR, serology and culture.

**Results:**

While side-effects associated with the collars were not noted, persistent fever necessitating enrofloxacin therapy occurred in two of the untreated cats. While *B. henselae* infection was ultimately confirmed in 4 of 7 of the untreated cats, none of the cats with collars became infected (P = 0.026).

**Conclusions:**

In this study design, use of a collar containing 10% imidacloprid and 4.5% flumethrin was well tolerated and prevented *C. felis* transmission of *B. henselae* amongst cats for 8 months.

## Background

*Bartonella henselae* is transmitted amongst cats by *Ctenocephalides felis* and is associated with multiple clinical syndromes in cats and people [1-7]. In addition, *Bartonella koehlerae* and *Bartonella clarridgeiae* have commonly been cultured or amplified from fleas, cats, and people. Being scratched by a young cat is a major risk factor for development of bartonellosis in people. *Bartonella henselae* is passed in *C. felis* frass and lives outside the body for 9 days or longer [8]. It is possible that the combination of *C. felis* and the grooming behavior of cats leads to *Bartonella* spp. contamination of claws. In one study of cats housed in shelters in Florida and Alabama with high risk for *C. felis* infestation, DNA of *Bartonella* spp. was amplified from the claws of 9 of 51 cats (17.6%) [6].

Use of flea control products that lessen transmission of *Bartonella* spp. amongst cats by *C. felis* may indirectly lessen the risk of bartonellosis to humans. In a previous study, monthly spot-on administration of 10% imidacloprid/1% moxidectin^a^ was shown to block transmission of *B. henselae* amongst cats experimentally exposed to infected *C. felis*[7]. However, this product is licensed for monthly use and so owner compliance may be less than 100%. A flea and tick collar containing 10% imidacloprid and 4.5% flumethrin^b^ was recently registered in the European Union and the United States [9-11]. This collar has been shown to be safe for use in kittens > 10 weeks of age and is very effective for the prevention of fleas and ticks for 8 months which could be very convenient for some cats and their owners [9-11].

The purpose of this study was to determine whether application of a flea and tick collar containing 10% imidacloprid and 4.5% flumethrin to cats would lessen *C. felis* transmission of *B. henselae* amongst cats over an 8 month study period.

## Methods

### *Bartonella henselae isolate*

*Bartonella henselae* strain CSU-1 was initially isolated from a shelter cat in Florida and has been passaged once in cats [7]. Blood in EDTA from a cat infected with CSU-1 by exposure to infected *C. felis* had been stored at −80°C until used in the current study.

### *Ctenocephalides felis*

The *C. felis* used in this study were purchased from an insectary of a local research laboratory.^c^ Two groups of 5 male and 5 female adult *C. felis* that were not exposed to the cats were purchased (one group before the study began and another group after the completion of this study) and were pooled by group, subjected to total DNA extraction, and shown to be negative for DNA of *Bartonella* spp. and haemoplasmas by PCR assay [6,11,12].

### Flea and tick collar

A flea and tick collar that contains imidacloprid 10% and flumethrin 4.5% was placed on designated cats in this study following the manufacturer’s guidelines [9-11].^b^The collars were stored in a locked, fireproof cabinet and maintained at 20°C to 25°C until used.

### Animals

A total of 19 cats (10 male; 9 female) were purchased from a commercial research cattery at approximately 4 months of age and shipped to the study site. The cats had been vaccinated against feline viral rhinotracheitis, feline calicivirus, and feline panleukopenia virus. During the 49 day equilibration period for the study, the male cats were neutered following protocols of the animal facility, all cats were shown to have negative serum test results for FeLV-antigen, FIV-antibody, *Dirofilaria immitis* antigen,^d^ and *Bartonella* spp. antibody (ELISA), and all cats were shown to be negative for *Bartonella* spp and haemoplasma DNA in blood using previously reported PCR assays [12-14]. A temperature sensing microchip was implanted as previously described for use during clinical monitoring over the course of the study [15].

### Experimental design

The study design was approved by the Institutional Animal Care and Use Committee at the research facility^e^ that housed the cats during the flea infestation portion of the study and the Animal Care and Use Committee at Bayer Animal Health.

After arrival at the facility and entry into the 49 day equilibration period, the cats were randomized into 3 groups and housed together in 1 room that was divided into 3 sections (R1, R2, and R3) beginning on Day −44. The 3 sections were separated from each other by mesh so that *C. felis* could move amongst the cats, while body contact between cats or fighting with members of other groups was prevented. Sections R1 and R3 were adjacent to the center section R2 but not to each other. Approximately 25% of the floor space in each section was covered in carpet to promote survival and a complete life cycle of *C. felis* within the room. Cat perches were placed in the corners of each section adjacent to the mesh dividing the sections to encourage cats from the various groups to be close to each other. Based on this design, it seems unlikely the physical structure itself would influence *C. felis* to preferentially transfer to R1 or R3.

The group in R1 (n = 7 cats) were untreated control cats throughout the study, the group in R2 (n = 4 cats) were infected with *B. henselae* by intravenous inoculation of 0.2 ml of blood containing *B. henselae* strain CSU-1 on Day – 39 of the study, and the flea and tick collars were placed on the group in R3 on Day 0 of the study. After placement of the collars on Day 0, a total of 50 males and 50 female *C. felis* were placed on each of the 4 cats in the *B. henselae* infected group in R2. Additional fleas (50 males and 50 female) were placed on each of the R2 cats monthly for an additional 6 applications and then every 12 – 14 days for 4 applications. The interval between *C. felis* application was shortened at the end of the experiment to increase likelihood of *B. henselae* infection in all cats. Flea counts by flea comb were determined every 12 – 14 days for the duration of the study and the fleas returned to the cat. The flea collars were removed from R3 cats on Day 238 of the study, all cats were administered imidacloprid^f^ once at that time, and then were observed and sampled as described until Day 254. Once shown to be negative for *B. henselae* by PCR, the cats were returned to the research facility cat colony with biosafety committee approval.

Samples were collected for performance of *Bartonella* spp. serology and PCR assay for amplification of *Bartonella* spp. DNA on Days 0, 14, 28, 42, 56, 70, 84, 98, 112, 126, 140, 154, 168, 182, 196, 210, 224, 238, and 252 for all cats as well as on Days −30, -23, -16, -9 and −2 for cats in R2.

### Assays

Blood samples (2 ml in a clot tube for serum separation; 1 ml in EDTA) from each cat were transported from the research facility to Colorado State University within 2 hours of collection. Samples were prepared for determination of serum titers of IgG against *Bartonella* spp. and for PCR assay for *Bartonella* spp. DNA on the day of collection [12,14]. One aliquot of each blood sample (500 μL) in EDTA was stored at −80°C for bacterial culture, pending results of the *Bartonella* spp. PCR assay. Samples negative for *Bartonella* spp. DNA by PCR assay were cultured using the stored blood as previously described [7]. Results for samples that yielded characteristic *Bartonella* spp. colonies were confirmed by *Bartonella* spp. PCR assay.

### Clinical monitoring

The cats were examined daily for attitude and appetite throughout the study. Mucous membrane color assessment and physical examination (including cardiac auscultation) was made on any cat exhibiting signs of depression or inappetance and for cats with a rectal body temperature of > 102.5°F (39.2°C). Any cat with fever had a complete blood cell count performed. Cats that developed clinical illness consistent with bartonellosis (fever and inappetance of > 2 days duration) during the study were administered enrofloxacin^g^ at 5 mg/kg, PO, daily for at least 14 days and supportive care as indicated. Cats requiring antibiotic treatment were administered imidacloprid^f^ and moved to a *C. felis* free room for continued care.

### Cat hair sampling

On Day 224, approximately 0.5 g of hair was collected from each cat. Each sample was placed into an individually labelled plastic bag for determination of imidacloprid concentrations.^h^ The cutoff sensitivity for the imidacloprid assay is 0.1 mg/kg hair.

### Statistical analysis

Cats were diagnosed with *B. henselae* infection if at least 2 samples over the course of the study were positive for *Bartonella* spp. IgG, *Bartonella* spp. DNA by PCR assay, or *Bartonella* spp. organisms by culture. The proportion of *B. henselae*-infected cats was compared between groups R1 and R3 by use of a 2-tailed Fisher exact test. Differences among group mean flea counts were compared with the 2-tailed Student *t* test. A value of *P* < 0.05 was considered significant for all analyses.

## Results

### Clinical findings

Side-effects associated with the collars were not noted in any cat throughout the study. While none of the cats in R2 or R3 developed clinical signs of bartonellosis after starting the *C. felis* infestations, 2 cats in R1 developed clinical signs necessitating treatment. For one cat from group R1, clinical signs were first detected on Day 47 of the study; the cat was normal 3 days after starting enrofloxacin therapy (14 days total) and fluids subcutaneously (once). The complete blood cell count for this cat was normal. The infection status of this cat was monitored in a separate room for several months. As the cat was still positive for *B. henselae* DNA on Day 154 and had only been administered imidacloprid once, over 100 days previously, it was returned to R1 to provide a continued source of *B. henselae* to attempt maximize the challenge infection for other cats in R1 and the cats in R3.

One other cat from group R1 developed clinical signs of bartonellosis on Day 124. Fever, lethargy, and a II/VI systolic left base heart murmur were the most significant findings. The complete blood cell count was normal other than the presence of 200 band neutrophils/μl (normal = 0 cell/μl) which could suggest inflammation. A scanning echocardiogram showed normal valve leaflets and so the cardiac murmur was believed to be from stress. The cat was negative for *Bartonella* spp. IgG but positive for *B. henselae* DNA by PCR assay on Day 124. The fever and lethargy resolved over the first 3 days of enrofloxacin administration (28 days total) and the heart murmur was not noted on subsequent recheck examinations. The cat was positive for *Bartonella* DNA alone on Days 154 and positive for *Bartonella* DNA and IgG on Day 168 and Day 182. This cat was not returned to the study.

### Assay results

All 4 of the group R2 cats became seropositive and PCR assay positive after IV inoculation. *Bartonella henselae* DNA was amplified from all 4 cats from Day 0 – 28 of the study. From Day 42 to Day 168, 1 – 3 cats in R2 were PCR positive on any given day. All R2 cats were PCR negative from Day 182 to Day 252. Each of the R2 cats seroconverted with maximal titers of 64; 128; 512; and 1028. The first positive antibody test results were detected on Day 56; Day 154 (2 cats), and Day 168. All 4 R2 cats were still seropositive on Day 252 (titers = 64; 64; 128; 256).

**Figure 1:**
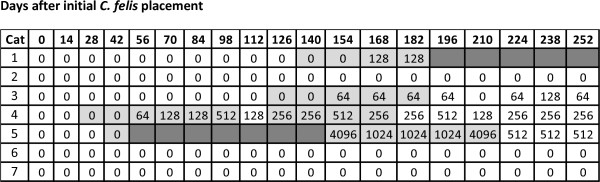
***Bartonella henselae *antibody and PCR assay results in 7 untreated cats (R1) exposed to *C. felis *allowed to feed on cats with *B. henselae *infection induced by IV inoculation.** Light gray shaded areas indicate samples that were PCR positive; Dark gray areas indicate the days on which the cats were removed from the room because of fever and need for supportive care; exposure to *C. felis* began on Day 0. None of the PCR negative cats were blood culture positive.

Of the 7 cats in R1 (no treatment), 4 cats had *B. henselae* amplified from blood with the first positive test results being detected on Days 28, 42, 126, and 140 (Figure 1). The first positive antibody test results for R1 cats were detected on Day 56; Day 154 (2 cats), and Day 168. Maximal titers were 64; 128; 512; and 4,096. None of the PCR negative cats were blood culture positive.

While *B. henselae* infection was ultimately confirmed in 4 of 7 of the untreated cats, none of the R3 cats with collars became infected (P = 0.026).

### Ctenocephalides felis counts

**Figure 2:**
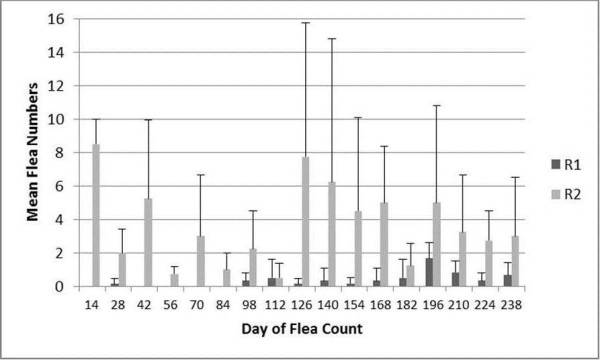
**Results (mean and standard deviation) of *Ctenocephalides felis *counts over time after placement of a flea collar on cats housed in R3 which was adjacent to R1 and R2.** The 4 cats in R2 had been experimentally infected with *Bartonella henselae* and a total of 50 males and 50 female *C felis* were placed on the R2 cats on Day 0, monthly for an additional 6 applications and then every 12 – 14 days for 4 applications. There were 6 – 7 cats in R1 over the course of the study. Only one *C. felis* was found on one cat in R3 over the course of the study (Day 238). The only day that *C. felis* counts were significantly different amongst groups was on Day 14 when cats in R2 had greater numbers of *C. felis* than cats in R1 or R3.

Most group R2 cats had detectable infestations when *C. felis* counts were made over time (Figure 2). However, the number of *C. felis* per cat varied on a given day. Only one *C. felis* was found on one cat in R3 over the course of the study (Day 238). The *C. felis* counts were variable for the cats in R1 as well. The only day that *C. felis* counts were significantly different amongst groups was on Day 14 when cats in R2 had great numbers of *C. felis* than cats in R1 or R3.

### Cat hair sampling

Imidacloprid could be detected in the hair samples of all group R3 cats, some of the R1 cats (4 of 6) and some of the R2 cats (1 of 4). The values varied amongst group R1 (mean = 0.25 mg/kg hair; SD = 0.22; range = 0 – 0.62), R2 (mean = 0.04 mg/kg hair; SD = 0.07; range = 0 – 0.15), and R3 (mean = 32.4 mg/kg hair; SD = 7.93; range = 17.89 – 41.71).

## Discussion

In this setting, use of a collar containing 10% imidacloprid and 4.5% flumethrin was well tolerated and prevented *C. felis* transmission of this strain of *B. henselae* amongst cats. These results are similar to those achieved in a previous study using 10% imidacloprid/1% moxidectin applied topically every month [7]. A potential limitation of the current study was the relatively low flea burdens on the cats in R1 detected over the course of the study which may have lessened the potential for transmission of *B. henselae* among the cats in this group. In the previous study, 100% of the control group developed *B. henselae* infection within the 3 month study period in contrast to a 57.1% infection reported here. The relatively low flea numbers on cats in R1 and R2 may also have lessened the risk for acquiring *B. henselae* infection in the cats with collars in R3.

The previous study of 10% imidacloprid/1% moxidectin applied topically every month used essentially the same study design [7]. However, in that study mean *C. felis* numbers on the control cats were generally > 2 fleas/cat after the first 14 days of the study which was numerically higher than the *C. felis* numbers reported in the control cats (R1) described here. Day 14 was the only time of the study described here that showed statistically higher *C. felis* counts in cats housed on R2 when compared to those in R1 and R3. This finding persisted even when the *C. felis* infestations were increased to every 14 days for the last month of the study. One likely explanation for these findings is that imidacloprid was shared amongst cats in the three groups by contact through the mesh walls. This hypothesis was the reason for collection of hair samples at day 224 for evaluation of their imidacloprid content. As imidacloprid is a very efficacious active ingredient, even low doses could interfere with flea survival on the animals and via killing flea larvae also interrupt the establishment of a constant flea population. Imidacloprid values found in the treated cat’s hair fit to the values detected in pre-licensing studies (Unpublished Data, 2012). However, traces of imidacloprid being at up to approximately 1% of this amount were also detected in the hair samples of untreated cats from R1 and R2. While only low concentrations of imidacloprid were detected, this was likely enough to lessen *C. felis* numbers, especially by interfering with the flea development life cycle.

At least one cat was *B. henselae* infected until Day 210 of the study and so could have served as a source of *B. henselae* for the new *C. felis* added in the later weeks of the experiment. These results suggest that the collar prevented *B. henselae* infection to the cats in R3 for the entire 8 month study period.

Development of fever and lethargy in 2 of 7 cats (28.5%) was similar to what was reported in the previous 10% imidacloprid/1% moxidectin study [7]. The results suggest that the CSU-1 strain of *B. henselae* is pathogenic to some cats and that virulence was not changed by one passage through cats. As observed in the previous study, the IV inoculated cats in R2 did not develop clinical illness which suggests pathogenic factors may be provided by the vector [7]. The two clinically ill cats administered enrofloxacin had rapid clinical responses suggesting a treatment effect. However, bacteremia was not eliminated in cats administered either a 14 day or 28 day course of therapy. Future research on how to limit *B. henselae* bacteremia in cats is needed.

## Conclusions

Use of imidacloprid containing products in this model can block transmission of this strain of *B. henselae* among cats. The CSU-1 strain of *B. henselae* can cause clinical disease in cats, particularly when vectored by *C. felis*.

## Endnotes

^a^Advantage Multi, Bayer HealthCare LLC, Shawnee Mission, KS; ^b^Seresto Bayer Animal Health, Leverkusen, Germany; ^c^HESKA Corporation, Loveland, CO; ^d^SNAP®Triple, IDEXX Laboratories, Portland, ME; ^e^High Quality Research, Fort Collins, CO; ^f^Advantage, Bayer HealthCare LLC, Shawnee Mission, KS; ^g^Baytril, Bayer HealthCare LLC, Shawnee Mission, KS; ^h^Bayer Animal Health GmbH, Leverkusen, Germany.

## Abbreviations

PCR: Polymerase chain reaction; R 1: Room 1; R2: Room 2; R3: Room 3; SD: Standard deviation.

## Competing interests

While this was a Bayer Animal Health funded project, all aspects were performed under GLP and GCP like conditions.

## Authors’ contribution

MRL served as the consultant for the project, provided the infectious disease assays, was the interfacing person between the outside research facility and Bayer Animal Health, and wrote the first draft of the experimental design and the manuscript. DS at Bayer Animal Health provided the funding for the project, provided input on all stages of the project including the experimental design and manuscript, and provided the interface among Bayer Animal Health, the research facility, and Colorado State University. JRH, MB, and AM provided scientific input into the study design and performed all assays. All authors approved the final version of the manuscript.
